# Bone Marrow Progenitor Cell Therapy-Mediated Paracrine Regulation of Cardiac miRNA-155 Modulates Fibrotic Response in Diabetic Hearts

**DOI:** 10.1371/journal.pone.0060161

**Published:** 2013-04-01

**Authors:** Raj Kishore, Suresh K. Verma, Alexander R. Mackie, Erin E. Vaughan, Tatiana V. Abramova, Ito Aiko, Prasanna Krishnamurthy

**Affiliations:** Feinberg Cardiovascular Research Institute, School of Medicine, Northwestern University, Chicago, Illinois, United States of America; University of Cincinnati, United States of America

## Abstract

Diabetes is associated with a higher incidence of myocardial infarction (MI) and increased risk for adverse vascular and fibrogenic events post-MI. Bone marrow-derived progenitor cell (BMPC) therapy has been shown to promote neovascularization, decrease infarct area and attenuate left ventricular (LV) dysfunction after MI. Unlike vascular effects, the anti-fibrosis mechanisms of BMPC, specifically under diabetic conditions, are poorly understood. We demonstrated that intramyocardial delivery of BMPCs in infarcted diabetic *db/db* mice significantly down-regulates profibrotic miRNA-155 in the myocardium and improves LV remodeling and function. Furthermore, inhibition of paracrine factor hepatocyte growth factor (HGF) signaling *in vivo* suppressed the BMPC-mediated inhibition of miR-155 expression and the associated protective effect on cardiac fibrosis and function. *In vitro* studies confirmed that the conditioned media of BMPC inhibited miR-155 expression and profibrotic signaling in mouse cardiac fibroblasts under diabetic conditions. However, neutralizing antibodies directed against HGF blocked these effects. Furthermore, miR-155 over-expression in mouse cardiac fibroblasts inhibited antifibrotic Sloan-Kettering Institute proto-oncogene (Ski) and Ski-related novel gene, non-Alu-containing (SnoN) signaling and abrogated antifibrogenic response of HGF. Together, our data demonstrates that paracrine regulation of cardiac miRNAs by transplanted BMPCs contributes to the antifibrotic effects of BMPC therapy. BMPCs release HGF, which inhibits miR-155-mediated profibrosis signaling, thereby preventing cardiac fibrosis. These data suggest that targeting miR-155 might serve as a potential therapy against cardiac fibrosis in the diabetic heart.

## Introduction

Experimental and clinical studies have shown the potential benefits of bone marrow-derived progenitor cell (BMPC) therapy for cardiovascular diseases [1], [2], [3]. Paracrine cytokines and growth factors released from transplanted progenitor cells have been shown to modulate cardiomyocyte survival, angiogenesis, mobilization and activation of endogenous stem cells [4], [5], [6]. Despite well-defined role of BMPC-mediated vasculogenesis, the molecular mechanisms involved in the antifibrosis effects of BMPC-based therapy are poorly understood. MicroRNAs (miR, small noncoding RNAs) are key regulators of gene expression and therefore, influence the pathophysiology of cardiovascular diseases [7], [8], [9]. Several miRNAs in the myocardium are modulated after MI including those that have been implicated in the regulation of fibrosis like miR-21, miR-29, miR-30, miR-133 and miR-155 [8], [9], [10], [11], [12]. Therefore understanding mechanisms that could regress MI-induced fibrosis in a relevant disease model of cardiac fibrosis would serve as a springboard for developing strategies to prevent cardiac dysfunction and improve post-infarct prognosis.

Diabetic patients have a 2- to 5-fold increased risk of developing heart failure and higher incidence of ischemic heart disease [13], [14]. Interestingly, diabetes also negatively influences subsequent cardiac remodeling events post-MI [15], therefore accounting for increased mortality among diabetic patients. Although the underlying mechanism is poorly understood, cardiac fibrosis has been shown to be a major feature of diabetic heart failure [16]. Hyperglycemia-induced fibrogenesis may negatively affect cardiac structure and function playing a specific role in the pathophysiology of heart failure in diabetes [17], therefore, necessitating the development of new therapeutic targets to treat LV dysfunction and remodeling in the diabetic heart.

In this study, we demonstrate that administration of BMPC in diabetic (*db/db*) mice after MI regulates cardiac miRNAs. BMPC administration reduced the expression of profibrotic miR-155 and fibrosis response in the diabetic hearts. We further show that neutralizing hepatocyte growth factor (HGF) signaling, both *in vitro* and *in vivo*, inhibits miR-155 expression and Ski/SnoN signaling leading to aggravated fibrogenesis response.

## Materials and Methods

### Bone Marrow Progenitor Cell (BMPC) Isolation and Culture

Mouse BMPC isolation, *ex vivo* expansion and culture of BMPCs was performed as previously described [3], [18], [19]. In brief, bone marrow mononuclear cells collected from C57BLKS/J mice (Jackson Laboratories, Bar Harbor, ME) were fractionated by density-gradient centrifugation with Histopaque-1083 (Sigma) and seeded onto culture dishes coated with 5 µg/ml human fibronectin (Sigma). Cells were maintained in endothelial cell basal medium-2 (EBM-2, Lonza, Walkersville, MD) supplemented with endothelial cell growth supplement (EGM-2 MV, Lonza) and 5% fetal bovine serum (FBS). Cells were cultured at 37°C with 5% CO_2_ in a humidified chamber. After 4 days in culture, adherent cells were washed with PBS and further cultured for 3 days in fresh growth medium. These cells showed characteristics of spindle shaped Endothelial Progenitor Cells (EPCs; data not shown) in accordance with previously published methods [3], [18], [19].

### Preparation of BMPC Conditioned Media (BMPC-CM) and Enzyme-linked Immunosorbent Assay (ELISA) for Secreted HGF

To produce BMPC conditioned medium (BMPC-CM), 5×10^6^ cells were cultured for 48 hours in growth factor-free EBM-2 with 1% FBS. The conditioned medium was then collected, filtered with a 0.22 µm filter (Pall Corp., Ann Arbor, MI) to harvest cell-free solution and concentrated (10X) by centrifugation using Ultrafree filter membranes (Millipore). EBM-2 containing 1% FBS without supplements served as control medium. Release of HGF into the media was measured by quantitative ELISA using HGF immunoassay kit (R&D Systems, Minneapolis, MN) as per manufacturers instructions. At least three independent measurements were performed in duplicates. HGF secretions from mouse endothelial cell line SVECs cultured under similar conditions were used for comparison. HGF levels are depicted in Figure S1 (supporting information).

### Isolation and Culture of Adult Mouse Cardiac Fibroblasts

Cardiac fibroblasts (CFs) were isolated from hearts of 12-week-old C57BLKS/J mice, as previously described [20], [21]. Briefly, the explanted hearts from mice were perfused with PBS-EDTA for 10 minutes, cut into pieces and digested with enzyme solution containing collagenase type II, trypsin and α-chymotrypsin (Worthington Biochem., Lakewood, NJ) at 37°C for 1 h, until the tissue blocks disappeared. The supernatant was subjected to Percoll density gradient centrifugation. After 1 h in culture, non-adherent cells were removed and adherent cells were grown to confluence (∼3 days) in Dulbecco’s modified minimum essential medium (DMEM) supplemented with 10% newborn calf serum and penicillin and streptomycin (1% v/v). CFs were then trypsinized (TrypLE™ Express, Gibco®) and replated on tissue culture grade dishes in DMEM/M199 medium and maintained at 37°C in humid air with 5% CO_2_. Confluent cells were almost exclusively fibroblasts (>98% purity), as previously reported [20], [21]. The cells were morphologically homogeneous with typical bipolar configuration observed by inverse microscopy.

### Assessment of miR-155 Expression and Fibrogenesis

To determine the effects of BMPC-CM on cardiac fibroblast miR-155 expression and fibrosis signaling *in vitro*, we exposed cardiac fibroblasts to diabetic *milieu* to activate fibrogenesis signaling and evaluated the effect of conditioned medium derived from cultured BMPC. To replicate the *in vivo* context (diabetic *milieu*), cardiac fibroblasts were grown in serum-free normal glucose (control) medium [5.5 mM glucose] for 24 h and exposed to 25 mM glucose +10 ng/ml TGF-β1 (diabetic *milieu*) with BMPC-CM or control medium. To correct for hyperosmolarity, a separate set of cells were exposed to 5.5 mM glucose +19.5 mM mannitol (OC, osmotic control). For assessing miR-155 expression (24 hr after treatment), total RNA was isolated using miRNeasy kit (Qiagen) and miRNA was analyzed by Taqman MicroRNA assay kits (Applied Biosystems, Carlsbad, CA) in accordance with the manufacturer’s protocol. miRNA levels were normalized with RNU6B (U6 control) expression. To evaluate fibrogenic response, expression of collagen type I alpha 1 (Col1A1), collagen type 3 alpha 1 (Col3A1) and alpha-smooth muscle actin (α-SMA) in cardiac fibroblast after 48 hr of treatment was assessed by qRT-PCR or Western blotting.

### miR-155 Over-expression in Cardiac Fibroblasts (CF)

To enable detailed study of miR-155 effects on fibrosis-related genes in cardiac fibroblasts, we transfected cardiac fibroblasts with either pre-miR-155 (miR mimics) or negative control mimics. Cardiac fibroblasts were grown in DMEM media without antibiotics and transfected with either miRNA mimics or controls (30 nM, Applied Biosystems) using Lipofectamine RNAiMAX (Invitrogen) for 72 hrs as per the manufacturer instructions. Transfected cells were ∼95% viable (data not shown). miRNA was assessed by qRT-PCR. Pre-miR-155 transfection significantly increased miR-155 expression as compared to mimic control transfected cells (P<0.01; Figure S2, supporting information).

### siRNA-mediated Knockdown of HGF in BMPCs

After BMPCs were cultured for seven days, the cells were transfected with small interfering RNA (siRNA) against mouse *HGF* or scrambled siRNA (Qiagen). Using Lipofectamine RNAiMAX (Invitrogen), the siRNA at a final concentration of 30 nM in Opti-MEM media (Invitrogen) was transfected into BMPCs, according to the manufacturer’s instructions. After 48 hrs of transfection, HGF secretion from these cells was confirmed by ELISA. siRNA directed against HGF significantly suppressed HGF secretion *in vitro* in comparison with control siRNA transfected cells (P<0.01; Figure S3, supporting information).

### Myocardial Infarction and Intramyocardial Transplantation of BMPCs in Diabetic Mouse

All animal research in this study was conducted in accordance with the protocols approved by the Institutional Animal Care and Use Committee (IACUC) at Northwestern University (Chicago, IL). Ten-weeks-old male C57BLKS/J *db/db* (diabetic) mice and their normal littermate controls (*db*/*+* mice) were purchased from The Jackson Laboratories (Bar Harbor, ME). Baseline blood glucose levels (blood obtained by tail snip) were measured using One Touch Ultra2 blood glucometer (Lifescan Inc., Milpitas, CA). *db/db* mice exhibited marked hyperglycaemia compared with relative *db*/*+* controls (non-fasting average blood glucose level 387.2±24.4 vs. 140.8±10.4 mg/dL, p<0.01). Mice were subjected to myocardial infarction (MI) by permanent ligation of left anterior descending coronary artery (LAD) as described previously [3]. Immediately after LAD ligation, 1×10^6^ BMPCs in a total volume of 15 µL was injected intramyocardially into 3 different sites in the periinfarct area distal to the ligation of the artery. Control animals received a corresponding volume of normal saline.

### *In vivo* Neutralization of HGF by an Anti-HGF Antibody or Administration of Recombinant Mouse HGF Protein

To determine if the antifibrosis effect of BMPC treated mice is inhibited by blocking HGF signaling, a subset of mice that underwent MI surgery and BMPC transplantation were injected intraperitoneally with neutralizing goat anti-HGF antibodies (25 µg/kg b.w. per day for seven days; R&D Systems, Minneapolis, MN) or normal goat IgG for 7 days, post-MI. Antibody dose is based on previous published data [22], [23]. Mouse recombinant HGF protein (PeproTech, Rocky Hill, NJ ) 10 µg in PBS was injected intramyocardially into 3 regions in the border zone distal to the ligation of the coronary artery immediately after induction of the MI followed by intraperitoneal injection (500 ng/mouse/day for five days). Control animals received a corresponding volume of PBS.

### Echocardiography Analysis

Transthoracic two-dimensional M-mode echocardiogram was obtained using Vevo 770 (VisualSonics, Toronto, Canada) equipped with a 30 MHz transducer. Echocardiographic studies were performed before (baseline) and at 7 and 28 days post-MI on mice anesthetized with a mixture of 1.5% isoflurane and oxygen (1 L/min). M-mode tracings were used to measure end-systolic diameter (LVESD) and end-diastolic diameter (LVEDD) and percent ejection fraction (%EF) was calculated as described previously [3], [19].

### Cardiac Fibrosis Assessment

Histopathology studies were performed as described previously [3], [19]. The hearts were fixed with 10% buffered formalin and paraffin embedded. Cardiac fibrosis was quantified on Picrosirius red (Polyscience, Inc., Warrington, PA, USA)-stained sections. Fibrosis area (red) and total LV area was measured using NIH’s ImageJ software to determine percent fibrosis.

### Quantitative Reverse Transcription PCR (qRT-PCR)

qRT-PCR was performed as described previously [3], [19]. RNA was extracted from cells using RNeasy mini kit (Qiagen). Following cDNA synthesis, amplification was performed using Taqman 7300 (Applied Biosystems, Foster City, CA). Relative mRNA expression of target genes was normalized to the endogenous 18 s control or GAPDH gene.

### Protein Isolation and Western Blot Analysis

Protein isolation and Western blotting for cultured cells were performed as previously described [3], [19]. Briefly, cells were homogenized in lysis buffer (Cell Signaling Technology, MA, USA) containing 20 mmol/L Tris-HCl [pH 7.5], 150 mmol/L NaCl, 2.5 mmol/L sodium pyrophosphate, 1 mmol/L β-glycerophosphate, 1 mmol/L sodium orthovanadate, 1 µg/ml leupeptin, 1 mmol/L ethylenediaminetetraacetic acid [EDTA], 1 mmol/L ethylene glycol tetraacetic acid [EGTA], 1% Triton X-100 and protease inhibitors. Equal amounts of protein were separated by 10% SDS-PAGE and blotted onto polyvinylidene difluoride (PVDF) membranes (Bio-Rad, Hercules, CA). The blots were incubated with antibodies against SnoN (Santa Cruz, USA), Ski (Santa Cruz, USA), collagen I (Southernbiotech), α-SMA (Abcam), beta-actin (Cell signaling) and developed with an enhanced chemiluminescence detection system (Amersham, Piscataway, NJ).

### Statistical Analysis

All data are expressed as mean ± SEM. Differences between groups were assessed by an unpaired Student’s *t*-test for single comparisons or by ANOVA for multiple comparisons, with Bonferroni post-hoc test. Values of *P*<0.05 were considered statistically significant.

## Results

### Intramyocardial BMPC Transplantation Modulates Cardiac miRNAs

**Figure 1:**
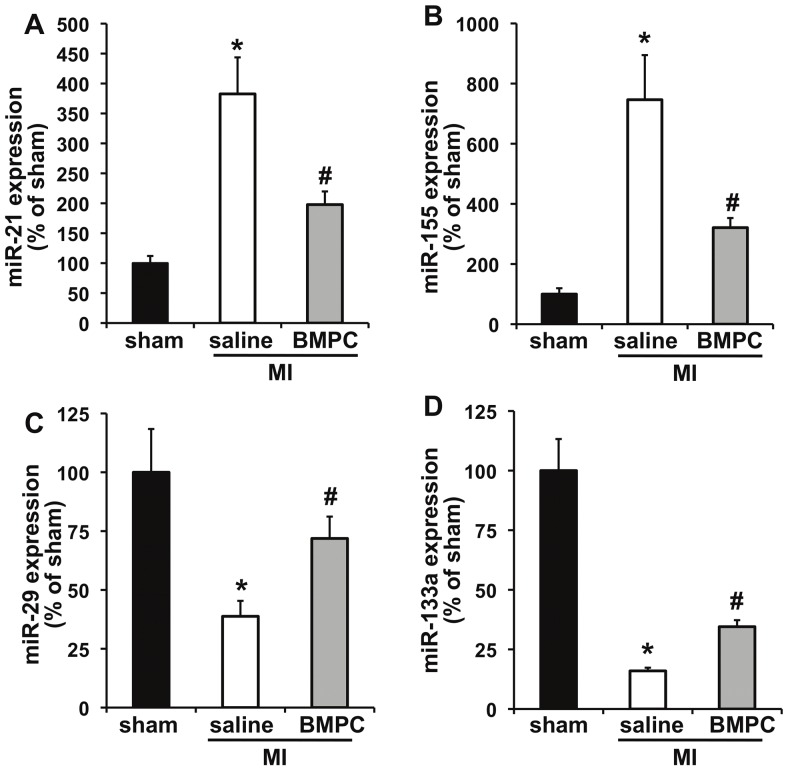
Intramyocardial BMPC transplantation regulates cardiac miRNAs following MI in mice. miRNA expression was measured in the border zone of infarcted area at 3 days post-MI by quantitative RT-PCR. BMPC therapy decreased miR-21 (**A**) and miR-155 (**B**) and increased miR-29 (**C**) and miR-133a (**D**) expression in comparison with saline-treated or sham groups. *P<0.01 vs sham; #P<0.05 vs saline group. BMPC-bone marrow-derived progenitor cell; MI, myocardial infarction.

To determine whether BMPCs regulate fibrosis-related miRNAs in infarcted heart, we injected mouse BMPCs in infarcted hearts of C57BLKS/J mice and determined (at 3 days post-MI) the expression of miRNAs (miR-21, miR-27, miR-29, miR-155, miR-30a and miR-133a, which have been shown to play a role in fibrosis [9], [10], [11], [24], [25]). Figure 1 depicts that saline-treated MI mice showed a significant increase in expression of miR-21 and miR-155 (P<0.01; Figures 1A and 1B) and decrease in miR-29 and miR-133a (P<0.01; Figures 1C and 1D) levels with non-significant reducing trend of miR-27 and miR-30a (Figures S4.A,B, supporting information). Interestingly, BMPC treatment inhibited MI-induced up-regulation of miR-21 (P<0.05; Figure 1A; shown to inhibit fibrosis by targeting sprouty homologue-1 [8] and phosphatase and tensin homologue [25]) and miR-155 (P<0.05; Figure 1B), which has been shown to play a role in cancer, atherosclerosis and immunomodulation [26], [27], [28], [29], [30]. Furthermore, BMPC administration upregulated miR-29 expression (P<0.05; Figure 1C), which is known to inhibit fibrosis by targeting collagen and fibrillin-1 [9] and increased miR-133a (P<0.05; Figure 1D), a negative regulator of connective tissue growth factor (CTGF) [10]. In contrast, the expression of miR-27 and 30a was not affected by BMPC therapy (Figure S4.A&B, supporting information). Together, these data indicate that BMPC administration modulates the expression of several fibrosis-related miRNAs after myocardial infarction.

The role of miRNAs −21, −29, −133a in fibrosis has been previously described [9], [10], [11], [24], [25]. However, miR-155 was most efficiently suppressed by BMPC treatment and its role in fibrosis is not known and has been shown to play an important role in acute viral myocarditis, cancer, inflammation and immunomodulation [26], [27], [28], [29], [30]. Recent study has shown that increased circulating miR-155 was predictive for cardiac death in post-AMI patients. [31]. Therefore, we further elucidated the significance of this miRNA in BMPC-mediated inhibition of fibrosis. We have previously shown that BMPC therapy reduced MI-induced cardiac fibrosis [3]. However, to evaluate its role in diabetes, we have tested the effect of BMPC on cardiac fibrosis in diabetic mice.

### BMPC Conditioned Media (BMPC-CM) Inhibits miR-155 Expression in Mouse Cardiac Fibroblasts (CFs) and Fibrogenesis Signaling in a Diabetic *milieu*, *in vitro*

**Figure 2:**
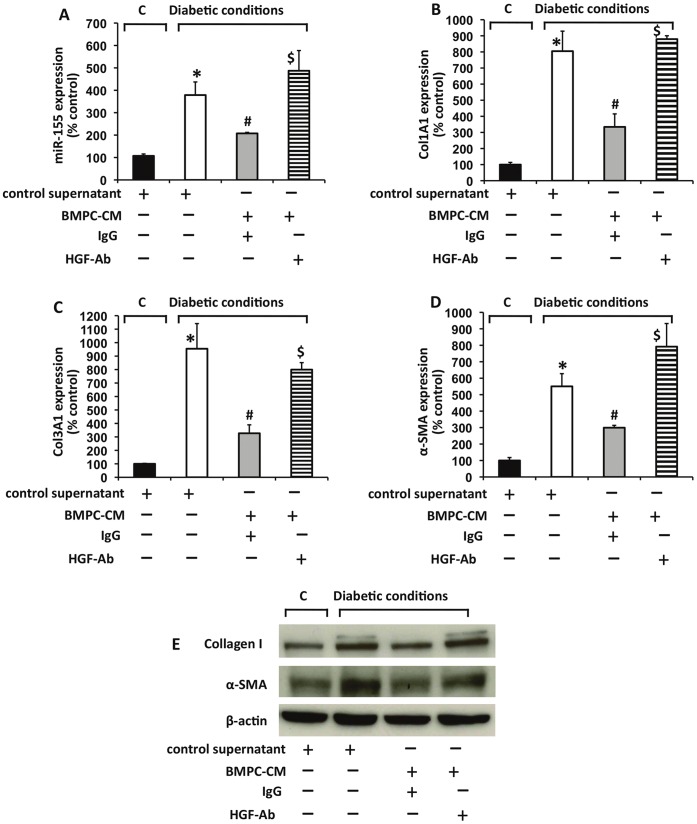
Conditioned media from BMPC regulates miR-155 expression and fibrogenic response in mouse cardiac fibroblasts *in vitro*. (**A**) conditioned media (CM) from BMPC reduced miR-155 expression (**A**) and mRNA expression of fibrogenesis markers like collagen type1 alpha 1(col1A1) (**B**), col3A1 (**C**) and α-SMA (**D**) in mouse cardiac fibroblasts induced under diabetic condition [TGF-beta (10 µg/mL+ high glucose (25 mM glucose)]. Neutralizing antibodies against HGF (HGF-Ab) reversed this effect as compared to IgG treated cells. *P<0.01 versus control; #P<0.05 versus without CM; $*P<*0.05 versus BMPC conditioned media with IgG. **E.** Protein expression of collagen I and α-SMA in cardiac fibroblasts with miR-155 over-expression was determined by Western blotting. β-actin served as loading controls. C indicates control condition (5.5 mM glucose); BMPC-CM, conditioned media from BMPC; control supernatant, control media; HGF-Ab, antibodies against hepatocyte growth factor; Col1A1, collagen type 1 alpha 1; Col1A1, collagen type 3 alpha 1; α-SMA, alpha smooth muscle actin.

Cardiac fibrosis significantly contributes to diabetes-induced diastolic dysfunction [9], [32], [33] and TGF-β activation plays a critical role in the process [34], [35], [36]. To determine whether BMPC may inhibit miR-155 expression in a paracrine manner, we evaluated the effect of conditioned medium derived from cultured BMPC on cardiac fibroblasts (CFs) exposed to diabetic *milieu* (to activate fibrogenesis signaling). Cardiac fibroblasts were grown in serum-free normal glucose (control) medium [5.5 mM glucose] for 24 h and exposed to 25 mM glucose +10 ng/ml TGF-β1 (diabetic conditions) with control or BMPC-CM. As shown in Figure 2A, diabetic conditions markedly increased the expression of miR-155 after 24 hours (P<0.01). In contrast, addition of BMPC conditioned media significantly suppressed the diabetic condition induced up-regulation of miR-155 (P<0.05). In association with increased miR-155, diabetic conditions significantly increased the mRNA expression of fibrotic markers, including Col1A1, Col3A1 and α-SMA after a 24 hour stimulation (Figures 2B, 2C and 2D; P<0.05) and protein expression of collagen I and α-SMA (Figure 2E) in CFs.

Among several paracrine factors secreted, the release of VEGF, SDF-1, IGF-1, and HGF was profoundly increased in EPC cell culture supernatants [4], [5]. HGF has been shown to play an important role in the pathogenesis of fibrotic cardiovascular disease [37], [38] and myocardial protection against ischemia/reperfusion injury [39]. To identify the factor that mediate the protective effect of BMPC conditioned media, the antifibrotic cytokine hepatocyte growth factor (HGF), a paracrine factor of BMPC [5], [24], [40] was blocked by neutralizing antibodies. The effect of BMPC conditioned media on miR-155 expression was significantly reversed by a neutralizing antibody directed against HGF (P<0.05 vs IgG treated cells; Figures 2A). Furthermore, BMPC-CM significantly inhibited fibrotic response induced by diabetic conditions (Figures 2B, 2C, 2D; P<0.05 and Figure 2E). However, neutralizing antibodies against HGF significantly reversed the effects of HGF (Figures 2). These data suggest that HGF, which is secreted by BMPC, inhibits diabetic condition-induced miR-155 expression and blocks fibrogenesis activity of cardiac fibroblasts *in vitro*.

### HGF Inhibition Abrogates the Antifibrosis Effect of BMPC after Myocardial Infarction in Diabetic *db/db* Mice

**Figure 3:**
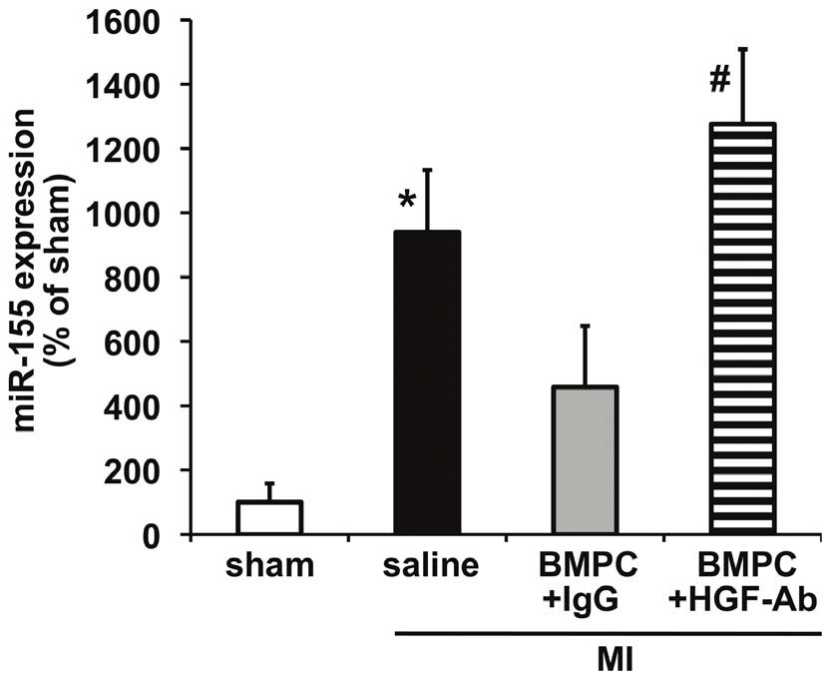
Expression of miR-155 in infarcted hearts (day 3) of *db/db* mice receiving BMPC intramyocardial. BMPC therapy marginally (although not significantly) decreased MI-induced miR-155 expression (measured by qRT-PCR) in comparison with saline-treated group. Administration of neutralizing antibodies against HGF (HGF-Ab) significantly elevated miR-155 levels as compared to IgG treated group. *P<0.05 versus sham; #P<0.01 versus IgG treated mice. HGF-Ab indicates antibodies against hepatocyte growth factor; Col1A1, collagen type 1 alpha 1; Col1A1, collagen type 3 alpha 1; α-SMA, alpha smooth muscle actin; BMPC, bone marrow-derived progenitor cell. BMPC, bone marrow-derived progenitor cell; MI, myocardial infarction. *P<0.01 vs sham; #P<0.05 vs IgG treated group.

**Figure 4:**
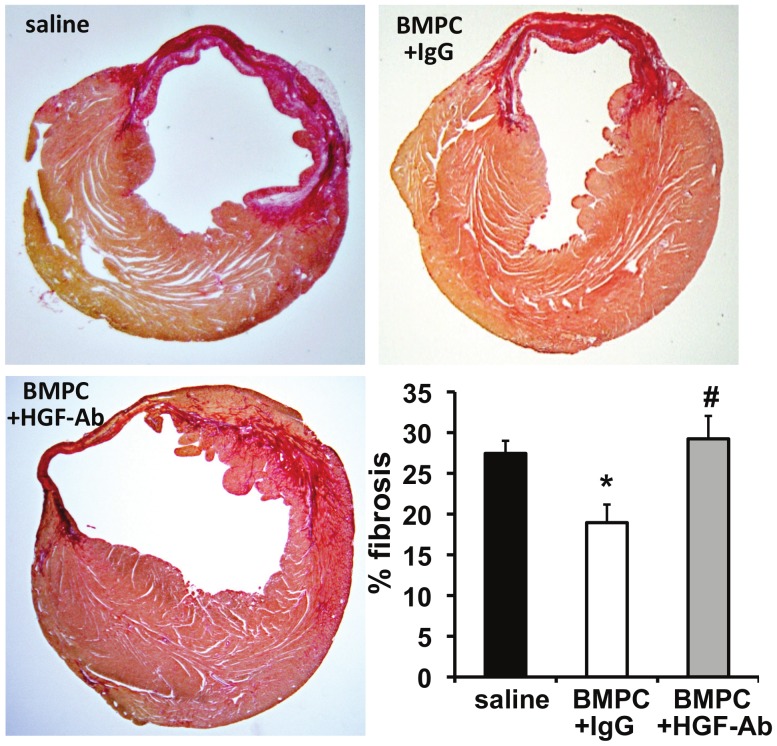
Cardiac fibrosis in *db/db* mice receiving BMPC therapy. Fibrosis area was determined at 28 days after MI by Sirius red staining. Collagen stains deep red and normal tissue stains yellowish. BMPC therapy decreased % fibrosis in comparison with saline-treated group. Administration of neutralizing antibodies against HGF (HGF-Ab) significantly aggravated fibrosis as compared to IgG treated group. Bar graph represents quantification of percent fibrosis area (percentage of LV area). HGF-Ab indicates antibodies against hepatocyte growth factor; BMPC, bone marrow-derived progenitor cell. BMPC, bone marrow-derived progenitor cell. *P<0.05 vs saline group; #P<0.05 versus IgG treated group.

**Figure 5:**
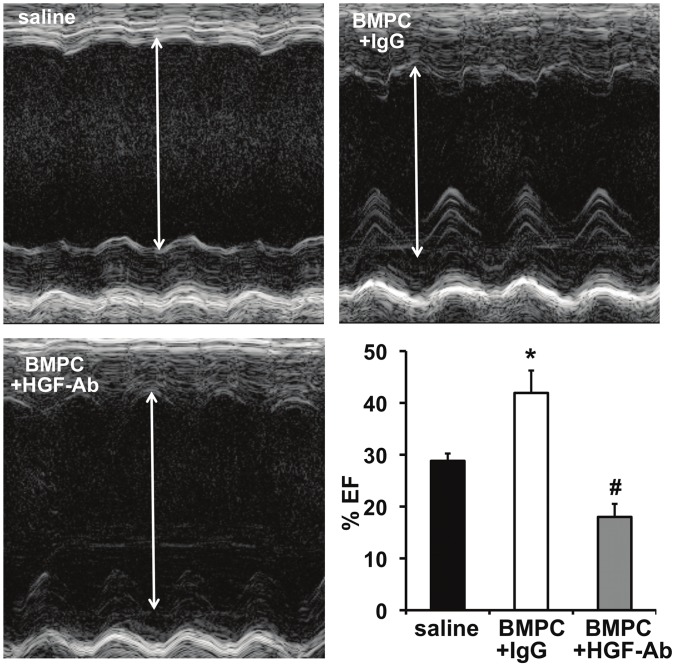
Echocardiographic analysis of LV function in *db/db* mice receiving BMPC therapy after 28 days post-MI. Ejection fraction (EF) increased following BMPC therapy (improvement in cardiac function) in comparison with saline-treated group. Administration of neutralizing antibodies against HGF (HGF-Ab) worsened LV function as compared to IgG treated group. *P<0.05 vs saline group; #P<0.05 versus IgG treated group. HGF-Ab indicates antibodies against hepatocyte growth factor; BMPC, bone marrow-derived progenitor cell. BMPC, bone marrow-derived progenitor cell.

**Figure 6:**
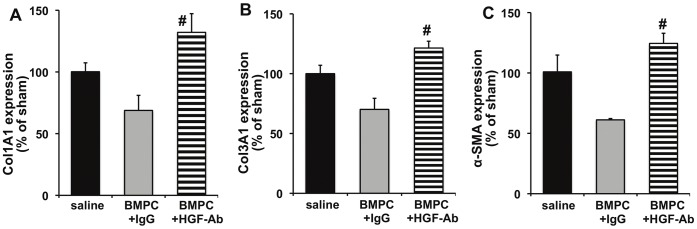
Expression of fibrotic markers (at 28 day post-MI) in infarcted hearts of *db/db* mice receiving BMPC intramyocardial. BMPC therapy reduced MI-induced mRNA expression (measured by qRT-PCR) of Col1A1, Col3A1 and α-SMA in comparison with saline-treated group. Administration of neutralizing antibodies against HGF (HGF-Ab) significantly aggravated fibrotic response as compared to IgG treated group. #P<0.05 versus IgG treated group. HGF-Ab indicates antibodies against hepatocyte growth factor; Col1A1, collagen type 1 alpha 1; Col1A1, collagen type 3 alpha 1; α-SMA, alpha smooth muscle actin; BMPC, bone marrow-derived progenitor cell. BMPC, bone marrow-derived progenitor cell.

Given the strong correlation between diabetes and cardiac fibrosis [13], [14], [15], [16], we explored whether BMPC therapy (via HGF secretion) inhibits miR-155 expression and prevents cardiac fibrosis in the infarcted hearts of diabetic mice. We transplanted BMPCs intramyocardially after MI followed by systemic administration of anti-HGF antibodies to neutralize secreted HGF *in vivo* in *db/db* mice. As compared to saline treated mice, BMPC transplantation reduced miR-155 expression (albeit not significant, Figure 3) in the myocardium at 3 days after MI and showed decrease in cardiac fibrosis (Figure 4; P<0.05 vs saline-treated mice) and improved LV function (increased %EF; Figure 5; P<0.05 vs saline treated mice) at 28 days after MI. Similarly, mRNA expression of ECM proteins, such as collagen (Col1A1, Col3A1) and α-SMA was increased in correlation with fibrotic response (Figure 6). Interestingly, HGF inhibition using anti-HGF antibodies in BMPC treated mice showed pronounced elevation of miR-155 expression (Figure 3, P<0.05 vs IgG-treated mice) in association with increased myocardial fibrosis (Figure 4; P<0.05), depressed cardiac function (reduced %EF; Figure 5; P<0.05) and increased mRNA expression of fibrotic markers (Figure 6) as compared to IgG treated mice. Together, these data demonstrate that BMPC-derived HGF regulates cardiac miR-155 and therefore modulates fibrosis in the infarcted heart.

Additionally, in a subset of mice, administration of recombinant HGF decreased miR-155 expression and reduced fibrosis and improved LV function (Figure S5, P<0.05 vs saline-treated mice). Furthermore, to elucidate the role of BMPC-derived HGF in regulating cardiac fibrosis, we inhibited HGF in mouse BMPC using siRNA and transplanted intramyocardially after MI in *db/db* mice. As compared to control siRNA BMPC treated hearts, inhibition of HGF in transplanted BMPC (siRNA-HGF) significantly increased miR-155 expression in the myocardium (Figure S6.A; P<0.05; supporting information) and aggravated cardiac fibrosis (Figure S6.B) and function (Figure S6.C), demonstrating that inhibition of HGF signaling reverses the effect of BMPC therapy on miR-155 expression and cardiac fibrosis and function in diabetic mice.

### miR-155 Over-expression Inhibits HGF-mediated Anti-fibrosis Effect in Mouse Cardiac Fibroblast, *in vitro*

**Figure 7:**
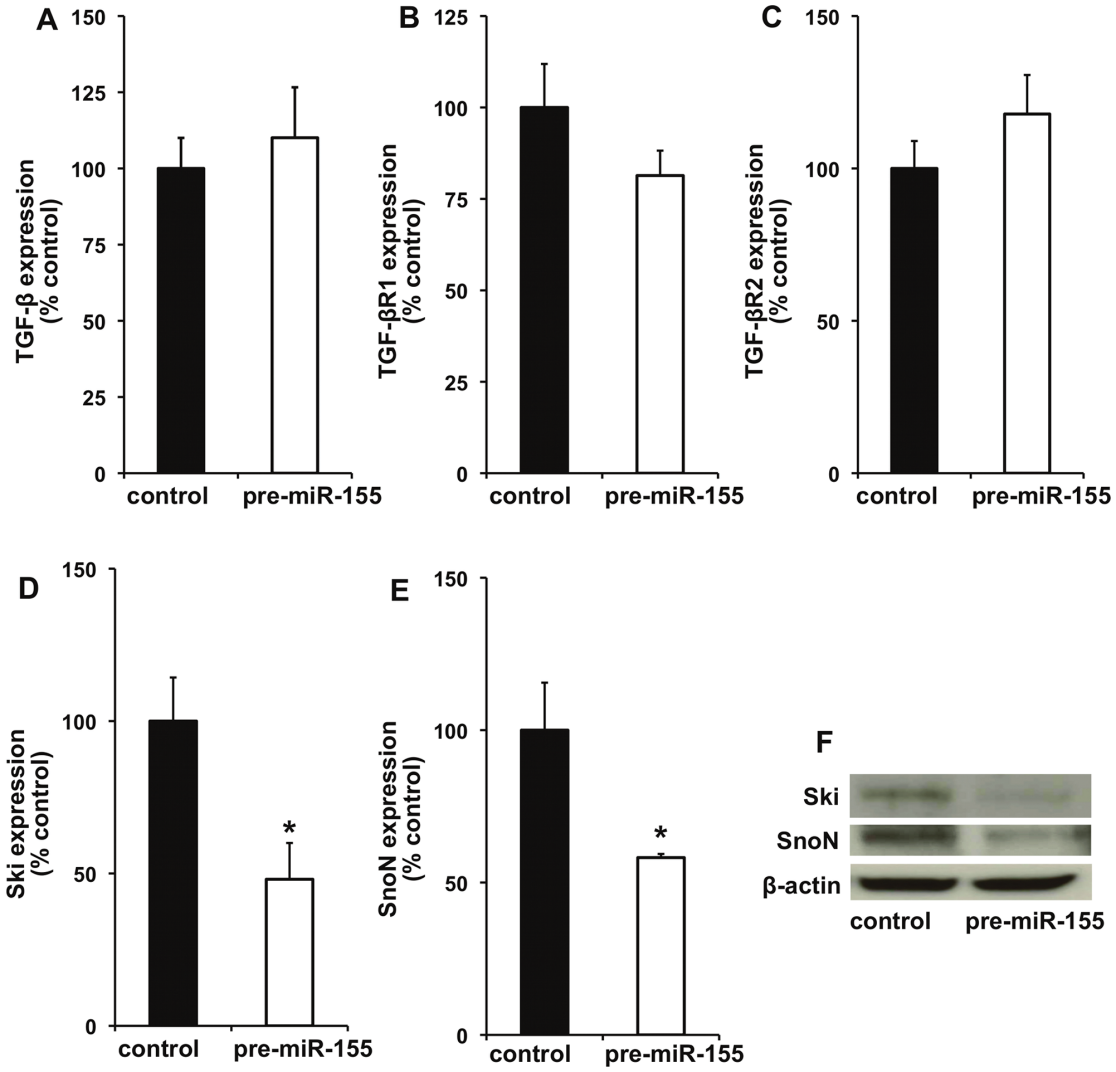
miR-155 over-expression modulates fibrosis-related signaling in mouse cardiac fibroblasts (CFs). CFs were either transfected with miR-155 mimics (pre-miR-155) to over-express miR-155 or negative control mimics (control) for 72 hrs and mRNA expression of various genes were determined by qRT-PCR. miR-155 over-expression (in presence of HGF) did not affect expression of TGF-β (**A**), TGF-βR1 (**B**) and TGF-βR2 (**C**). In contrast mRNA expression of Ski (**D**) and SnoN (**E**) was decreased. *P<0.05 versus control cells. **F**. Protein expression of Ski and SnoN in cardiac fibroblasts following over-expression of miR-155 was determined by Western blotting. β-actin served as loading controls. TGF-β indicates TGF-β; TGF-βR1, TGF-beta receptor type 1; TGF-βR2, TGF-beta receptor type 2; Ski, Sloan-Kettering Institute proto-oncogene; SnoN, Ski-related novel gene, non-Alu-containing.

**Figure 8:**
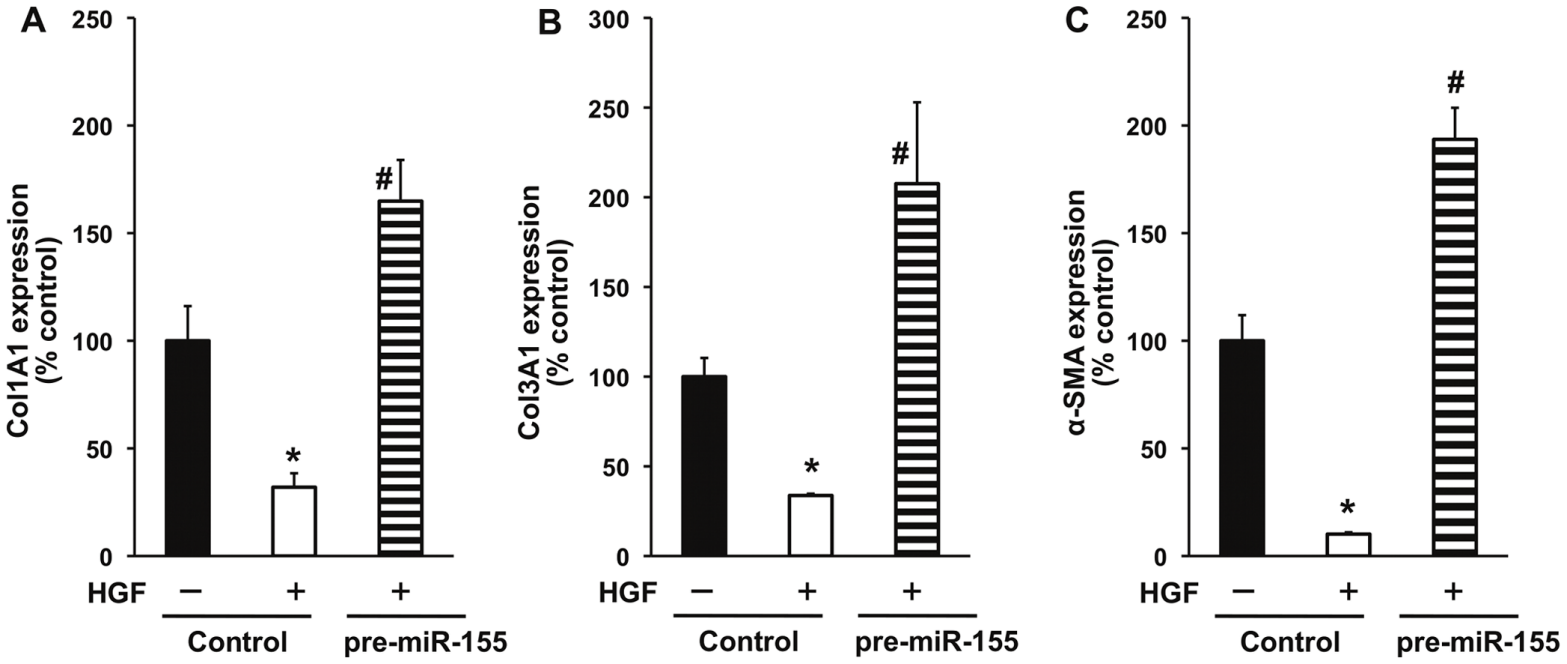
miR-155 over-expression abrogates HGF-induced inhibition of fibrogenic response in mouse cardiac fibroblasts (CFs). CFs were either transfected with miR-155 mimics (pre-miR-155) to over-express miR-155 or with negative control mimics (control), treated with or without HGF under diabetic conditions [TGF-β (10 ug/mL+high glucose (25 mM glucose)] and mRNA expression of fibrogenesis markers like col1A1 (**A**), col3A1 (**B**) and α-SMA (**C**) were measured by qRT-PCR. HGF decreased the expression of above markers. *P<0.01 versus without HGF. Over-expression of miR-155 nullified the effects of HGF. P<0.01 versus control-transfected cells. HGF indicates hepatocyte growth factor; Col1A1, collagen type 1 alpha 1; Col1A1, collagen type 3 alpha 1; α-SMA, alpha smooth muscle actin.

To evaluate the effect of miR-155 on fibrosis signaling, we over-expressed miR-155 in mouse cardiac fibroblasts using miR-155 mimic (pre-miR-155) and determined the effect on HGF-mediated anti-fibrosis signaling. As shown in Figure 7, miR-155 over-expression did not affect TGF β1, TGF-β receptor 1 (TGF-βR1) and TGF-β receptor 2 (TGF-βR2) (Figure 7A,B,C). We further evaluated miR-155 effect on inhibitors of TGF-β signaling like Sloan-Kettering Institute proto-oncogene (Ski) and Ski-related novel gene, non-Alu-containing (SnoN). Previous studies have shown that Ski/SnoN are expressed in all tissues at low levels and HGF up-regulates Ski/SnoN and therefore inhibits fibrosis response [41]. The Ski proto-oncoprotein has been shown to co-repress TGF-β1 post-receptor signaling through receptor-activated Smads (R-Smads) [41] and Ski inhibits fibrosis through regulation of cardiac myofibroblast phenotype [42]. Interestingly, miR-155 over-expression significantly decreased mRNA expression of Ski/SnoN (Figure 7D and 7E; P<0.05) and protein levels (Figure 7F) as compared to control cells. Next, we determined whether miR-155 over-expression affects HGF-mediated antifibrogenic response in cardiac fibroblasts under diabetic *milieu* (to stimulate fibrogenesis response). Interestingly, miR-155 over-expression abrogated HGF-mediated decrease in fibrosis-related markers like Col1A1, Col3A1 and α-SMA (Figure 8; P<0.01), suggesting that antifibrotic effect of HGF/miR-155 might be working predominantly through suppression of negative feedback inhibitor of TGF-β signaling.

## Discussion

**Figure 9:**
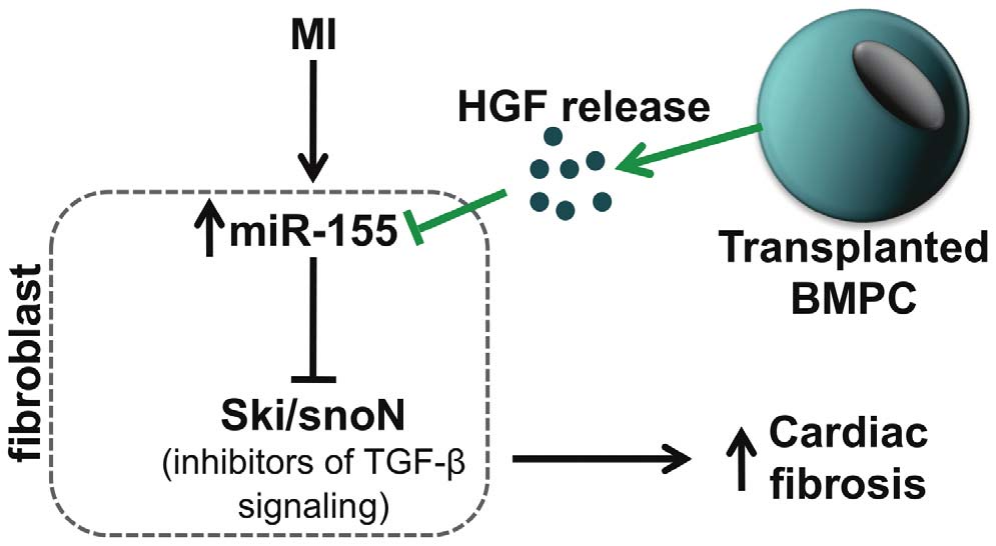
Proposed mechanisms of the paracrine regulation of cardiac fibrosis by transplanted BMPCs. BMPCs release HGF, which inhibits miR-155 leading to de-repression of inhibitors of TGF-β signaling, Ski/SnoN (possible targets of miR-155), thereby inhibiting cardiac fibrosis. HGF indicates hepatocyte growth factor; BMPC, bone marrow-derived progenitor cell. MI indicates myocardial infarction; BMPC, bone marrow-derived progenitor cell; HGF, hepatocyte growth factor; TGF-β, TGF-beta; Ski, Sloan-Kettering Institute proto-oncogene; SnoN, Ski-related novel gene, non-Alu-containing.

Cardiac fibrosis is a pathological hallmark of diabetic complications. In the present study we demonstrate that bone marrow-derived progenitor cell (BMPC) administration regulates expression of miRs (specifically miR-155) and modulates fibrosis in the infarcted heart in diabetic (*db/db*) mice. Furthermore, neutralization of paracrine factor, hepatocyte growth factor (HGF) aggravates fibrogenic response both *in vivo* and *in vitro* (possible mechanism is depicted in Figure 9). Experimental and clinical studies have shown potential benefits of bone marrow-derived cell therapy for cardiovascular diseases [1], [2], [6]. Among other mechanisms, paracrine cytokines and growth factors released from transplanted progenitor cells have been shown to modulate cardiomyocyte survival, angiogenesis, mobilization and activation of endogenous stem cells, resulting in the reduction of infarct size and improvement of the left ventricle function [4], [5], [24], [40], [43]. Although it is well accepted that BMPC mediates tissue repair through angiogenesis/vasculogensis, little is known about the molecular mechanisms underlying the anti-fibrotic response upon BMPC transplantation in the myocardium.

HGF (also known as “scatter factor”) is a multifunctional molecule that elicits a wide spectrum of biological activities in many patho-physiological processes [39], [44], [45]. In the heart, HGF has been shown to exert anti-apoptotic/cardioprotective effects in rats subjected to MI [39], [44] and promotes cardiac regeneration through its antioxidant effects [44]. Also, HGF is a potent angiogenic factor that mobilizes EPCs [45] and protects human endothelial cells against advanced glycation end product-induced apoptosis [46]. Despite the clear indications for HGF to effectively treat post-ischemic heart failure, knowledge on the mechanism of HGF signaling in fibrosis is still limited. In particular, nothing is known about the role of the HGF signaling in BMPC-based therapy for cardiac regeneration in diabetic hearts.

HGF has been shown to inhibit TGF-β mediated fibrosis signaling in a variety of disease models including renal and cardiovascular systems [37], [44], and specifically under diabetic conditions [46]. Our findings in the present study that inhibition of HGF secretion (HGF siRNA transfected BMPC) or neutralization of secreted HGF using antibodies against HGF *in vivo* abrogates the antifibrosis effect of BMPC therapy, suggesting that BMPC release of paracrine factor (HGF) suppresses profibrogenic signaling in diabetic hearts. In addition, we studied the effect of recombinant HGF protein administration on cardiac fibrotic response to account for any loss or marginalization of effect in BMPC transfected with HGF siRNA. Together, these data correlate with the previous study that demonstrated that *in vivo* gene transfer of HGF before ischemia showed multiple beneficial actions (specifically antioxidant effect), therefore contributing to the reduction in the infarct size and the improvement of left ventricular dysfunction after infarction [44]. Furthermore, supernatants of BMPC inhibit fibrogenesis response in cardiac fibroblasts, which was inhibited upon neutralization of HGF using antibodies against HGF. These data suggest that the antifibrosis effect of BMPC might be mediated through paracrine secretion of HGF. It should be noted that several other cytokines are known to influence cardiac function and may play important roles, particularly in pathologic situations such as acute myocardial infarction [4], [5]. Although, cytokines other than HGF were not examined in the present study, it is possible that HGF modulates the ischemia-reperfusion injury and post-infarct process through interaction with other cytokines. However, the mechanism of HGF-mediated antifibrosis effect, specifically in diabetes is poorly understood.

Diabetes is an important risk factor for coronary heart disease and contributes to the development of cardiovascular diseases. An increase in TGF-β production is commonly thought to contribute to excessive cardiac fibrosis in patients with diabetes [34]. Fibroblasts are responsible for the synthesis of Extra Cellular Matrix (ECM) components, both in the healthy heart, as well as in pathological fibrosis [34]. However, understanding signaling cascades that control ECM synthesis and degradation, and fibroblast proliferation and apoptosis are crucial in evolving stem cell-based strategies to inhibit cardiac fibrosis. We have previously shown that BMPC therapy reduced MI-induced cardiac fibrosis [3], [19]. Given that microRNAs (miRNAs) modulate pathophysiology of cardiovascular diseases through regulation of gene expression [7], [8], [9], [47], we determined whether BMPCs administration after MI regulates miRNAs (like miR-21, miR-27, miR-29, miR-155, miR-30a and miR-133a) that have been shown to play a role in fibrosis in various tissues/organs [8], [11], [12]. Saline-treated (control) MI mice showed a significant up-regulation of miR-21 and miR-155 and decrease in miR-29 and miR-133a expression (Figure 1). These results are consistent with the previous findings [8], [9], [25], [27], however it should be noted that several other miRNAs (related or unrelated to fibrosis) might have been modulated as reported by earlier investigators [7], [11]. Interestingly, BMPC administration modulates the expression of several fibrosis-related miRNAs after MI, specifically up-regulated miR-29 and miR-133a and down-regulated miR-155 and miR-21. However, the role of miRNAs −21, −29, −133a in cardiac fibrosis has been previously described [9], [10], [25]. For instance, miR-29 has been shown to inhibit fibrosis by targeting collagen and fibrillin-1 [9], miR-133a negatively regulates connective tissue growth factor (CTGF) [10] and miR-21 targets sprouty homologue 1 [8] and phosphatase and tensin homologue [25]]. Although the role miR-27 and miR-30a in fibrosis has been established in other organs systems [12], [48], their levels remained unchanged in the present study.

Interestingly, miR-155 was most efficiently suppressed by BMPC treatment in the present study. Several studies have reported that miR-155 expression is modulated in a number of physiological and pathological processes such as hematopoiesis, immunity, tumorigenesis [26], [27], [28], [29] and inflammation [49]. Recent study has shown that a subset of circulating miRNAs (specifically, increased serum miR-155 and miR-380) are predictive for cardiac death in human patients after hospital discharge for acute myocardial infarction [31]. In a mouse model of lung fibrosis, miR-155 has been shown to be increased in response to several inflammatory mediators in different cell types [50]. High levels of miR-155 were detected in synovial fibroblasts from rheumatoid arthritis patients, an autoimmune disorder associated with high inflammation [51]. TGF-β-treated murine mammary gland (NMuMG) epithelial cells showed significantly elevated miRNA-155 levels and knockdown of miR-155 suppressed TGF-β-induced epithelial-mesenchymal transition (EMT) [28]. Furthermore, inhibition of miR-155 attenuated cardiac inflammatory response and myocardial damage in acute viral myocarditis in mice [27]. Given that diabetes is associated with chronic inflammatory response; and that miR-155 plays an important role in cancer, inflammation and immunomodulation [26], [27], [28], [29] and limited literature is available regarding its role in cardiac fibrosis especially in the setting of diabetes and BMPC therapy, we further elucidated the significance of this miRNA in BMPC-mediated inhibition of fibrosis. Therefore, we next explored how HGF inhibited fibrosis after myocardial ischemia under diabetic conditions. Along with the progression of fibrosis, the expression of miR-155 was significantly elevated after MI in the hearts of *db/db* mice that received BMPC transfected with siRNA against HGF, as compared to control siRNA BMPC treated hearts. Similarly, mRNA expression of ECM proteins, such as collagen I, collagen III and α-SMA, was also significantly increased in correlation with fibrotic response (Figure 4b). Furthermore, inhibition of HGF signaling *in vivo* by systemically applying antibodies against HGF significantly elevated miR-155 expression and aggravated cardiac fibrosis as compared to BMPC-treated mice (Figure 6). Together, these data demonstrate that BMPC-derived HGF regulates cardiac miR-155 and therefore fibrosis in the infarcted heart. However, functions of miR-155 in cardiac fibroblasts and its role in fibrogenesis response have not yet been documented.

To explore the potential intracellular molecular mechanism by which miR-155 promotes fibrosis response in diabetes, we examined the effect of miR-155 over-expression in cardiac fibroblasts on TGF-β, TGF-β receptors -I & -II and co-repressor of TGF-β signaling like Ski/SnoN. Over-expression of miR-155 in cardiac fibroblasts only marginally affected the mRNA expression of TGF-β1 and its type I and type II receptors. However, miR-155 over-expression inhibited expression of Ski and SnoN at both mRNA and protein levels in cardiac fibroblast cells. Previous reports have shown that Ski/SnoN physically interacted with activated Smad-2 by forming transcriptionally inactive complex and overrode the profibrotic action of TGF-β1 [41]. Cellular transdifferentiation through Endothelial to mesenchymal transition (EndoMT) induced by TGF-β1 has emerged as a possible source of tissue myofibroblasts and cardiac fibrosis and HGF has been shown to inhibit EndoMT in the heart [38]. Previous reports have demonstrated that HGF blocks TGF-β1 signaling via up-regulating Smad transcriptional co-repressor SnoN expression [52]. It is not clear whether BMPC therapy specifically affects EndMT process in the present study. However, the findings in the present study illustrate the potential of confining Smad activity using anti-miR-155 as an effective strategy for blocking cardiac fibrosis. Also, Ski has been shown to be a direct target of miR-155 in melanoma cell lines [29]. Furthermore, computational analysis (using TargetScan database) shows that Ski lodges a complimentary sequence for miR-155. In the present study, miR-155 over-expression in cardiac fibroblasts significantly suppressed HGF-induced Ski expression, suggesting that Ski/SnoN might be a direct target of miR-155 and therefore exhibits profibrotic response through suppression of Ski/SnoN signaling.

Together, our data demonstrates that the paracrine regulation of cardiac miRNAs by transplanted BMPCs contributes to the antifibrotic effects of cell therapy. In particular, BMPCs release HGF, which inhibits the miR-155-mediated profibrosis response leading to inhibition of cardiac fibrosis and improvements in cardiac function. Based on these data and available literature, we anticipate that targeting miR-155 might serve as a potential therapy against cardiac fibrosis in diabetic heart.

## Supporting Information

Figure S1 **Secreted HGF levels.** ELISA for HGF secreted by BMPCs after 48 hrs in growth-free EBM-2 media. Secretion from SVEC in similar media was used for comparison.
(TIF)

Figure S2 **miR-155 over-expression in adult mouse cardiac fibroblasts.** miR-155 expression (determined by qRT-PCR) was increased in cardiac fibroblasts transfected with miR-155 mimics (pre-miR-155) for 72 h as compared to cells transfected with negative control mimics (control). *P<0.01 compared with control-transfected cardiac fibroblasts.
(TIF)

Figure S3 **ELISA for HGF levels secreted from BMPCs transfected with HGF siRNA.** HGF secretion was reduced in BMPCs 48 hours after transfection with siRNA directed against HGF. P<0.01 versus control siRNA transfected cells.
(TIF)

Figure S4 **Expression of cardiac miRNAs after intramyocardial BMPC transplantation following MI in mice.** miRNA expression was measured in the border zone of infarcted area at 3 days post-MI by quantitative RT-PCR. BMPC therapy did not affect miR-27 (**A**) and miR-30a (**B**) in comparison with saline-treated group. BMPC, bone marrow-derived progenitor cell; MI, myocardial infarction.
(TIF)

Figure S5 **Administration of mouse recombinant HGF provided cardiac protection after MI.** (**A**) HGF administration reduced miR-155 expression, enhanced LV function (increased % EF) (**B**) and inhibited fibrosis (**C**). *P value versus saline-treated MI mice.
(TIF)

Figure S6 **Transplantation of BMPC transfected with siRNA against HGF in mice after MI.** (**A**) miR-155 expression, percent ejection fraction (% EF) (**B**) and % fibrosis (**C**) in mice receiving intramyocardial BMPC transfected with siRNA against HGF after MI. *P<0.05 versus control siRNA BMPC-treated MI mice.
(TIF)
