# Supplementary figures and images for: Bone Marrow Progenitor Cell Therapy-Mediated Paracrine Regulation of Cardiac miRNA-155 Modulates Fibrotic Response in Diabetic Hearts
Source: PLoS One. 2013 Apr 1;8(4):e60161. doi: 10.1371/journal.pone.0060161 (PMC3613379; doi:10.1371/journal.pone.0060161)

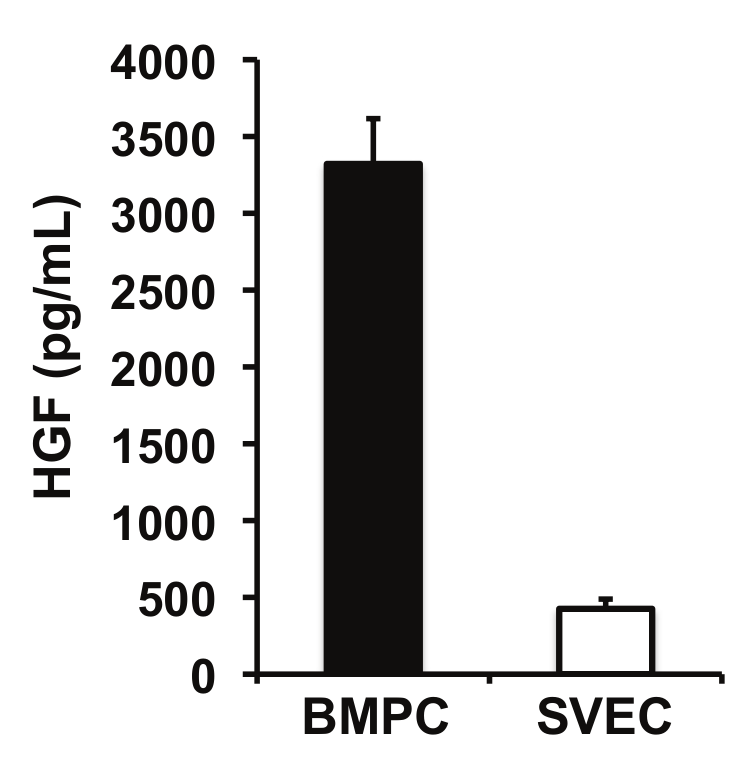

Supplement: Figure S1 — Secreted HGF levels. ELISA for HGF secreted by BMPCs after 48 hrs in growth-free EBM-2 media. Secretion from SVEC in similar media was used for comparison. (TIF) [file pone.0060161.s001.tif]

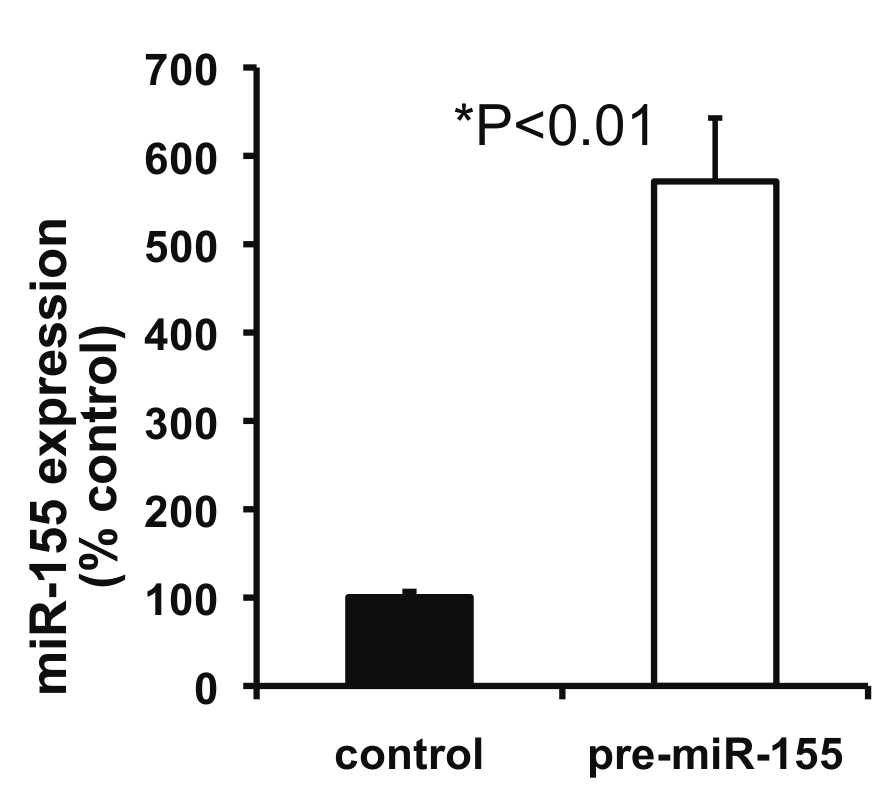

Supplement: Figure S2 — miR-155 over-expression in adult mouse cardiac fibroblasts. miR-155 expression (determined by qRT-PCR) was increased in cardiac fibroblasts transfected with miR-155 mimics (pre-miR-155) for 72 h as compared to cells transfected with negative control mimics (control). *P<0.01 compared with control-transfected cardiac fibroblasts. (TIF) [file pone.0060161.s002.tif]

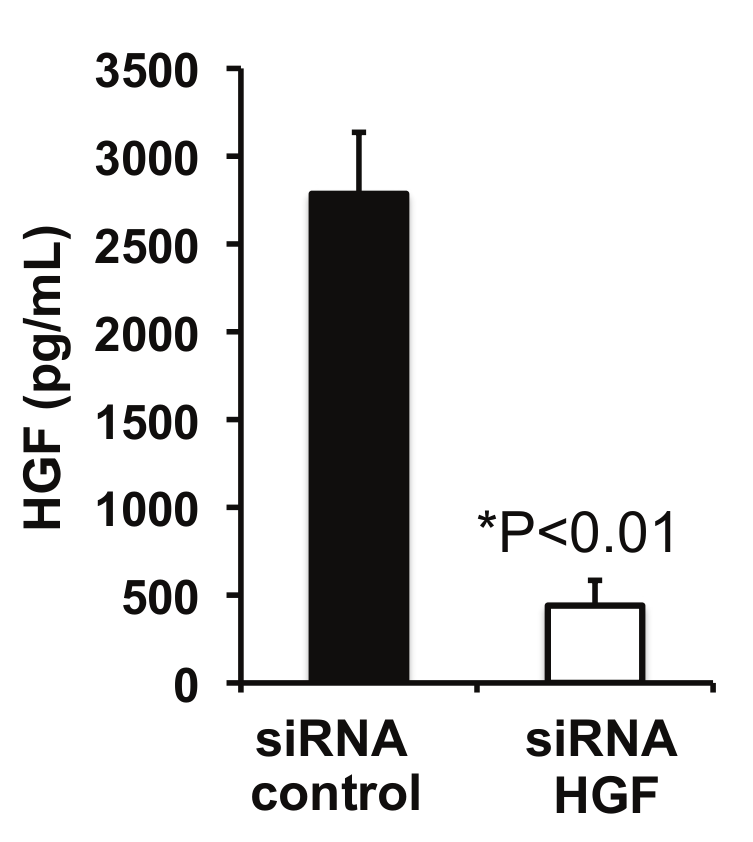

Supplement: Figure S3 — ELISA for HGF levels secreted from BMPCs transfected with HGF siRNA. HGF secretion was reduced in BMPCs 48 hours after transfection with siRNA directed against HGF. P<0.01 versus control siRNA transfected cells. (TIF) [file pone.0060161.s003.tif]

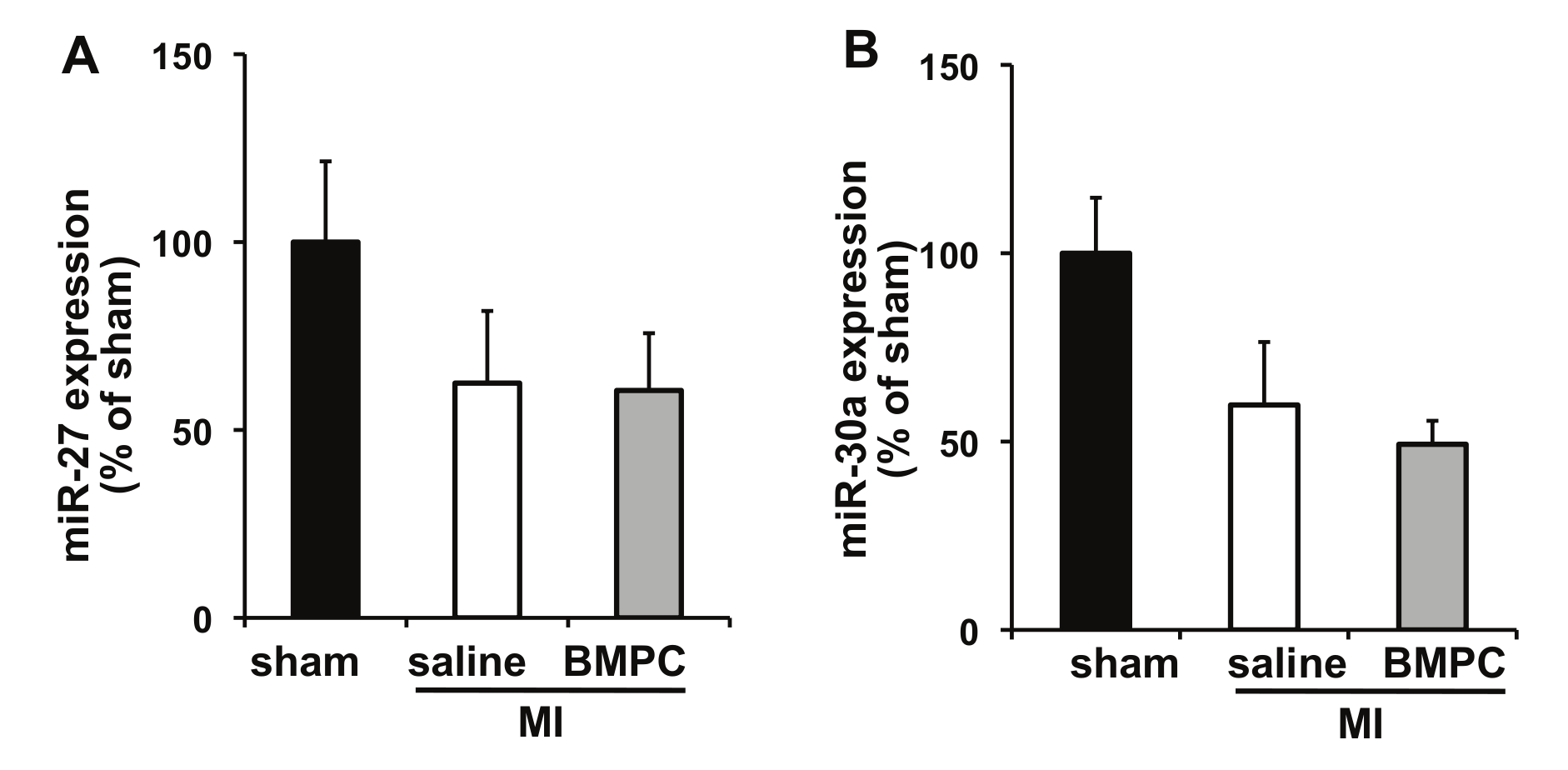

Supplement: Figure S4 — Expression of cardiac miRNAs after intramyocardial BMPC transplantation following MI in mice. miRNA expression was measured in the border zone of infarcted area at 3 days post-MI by quantitative RT-PCR. BMPC therapy did not affect miR-27 (A) and miR-30a (B) in comparison with saline-treated group. BMPC, bone marrow-derived progenitor cell; MI, myocardial infarction. (TIF) [file pone.0060161.s004.tif]

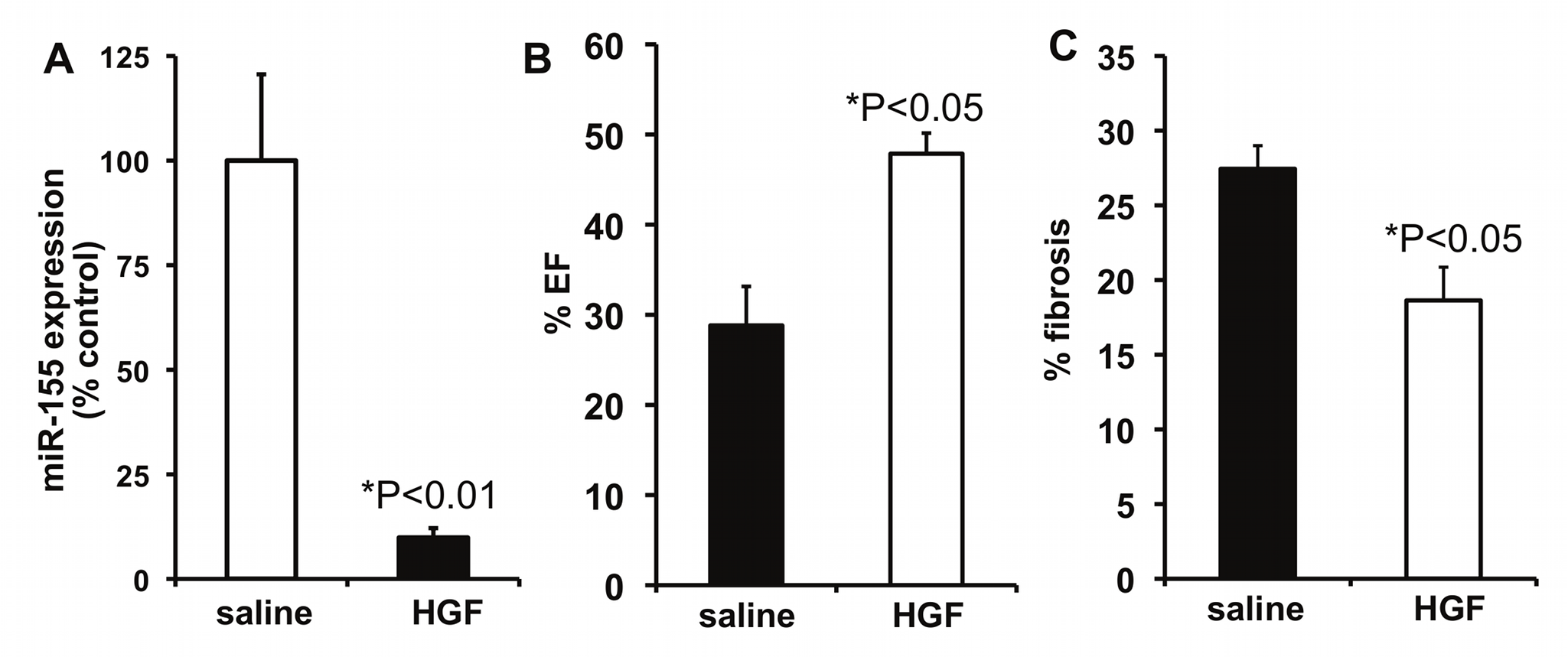

Supplement: Figure S5 — Administration of mouse recombinant HGF provided cardiac protection after MI. (A) HGF administration reduced miR-155 expression, enhanced LV function (increased % EF) (B) and inhibited fibrosis (C). *P value versus saline-treated MI mice. (TIF) [file pone.0060161.s005.tif]

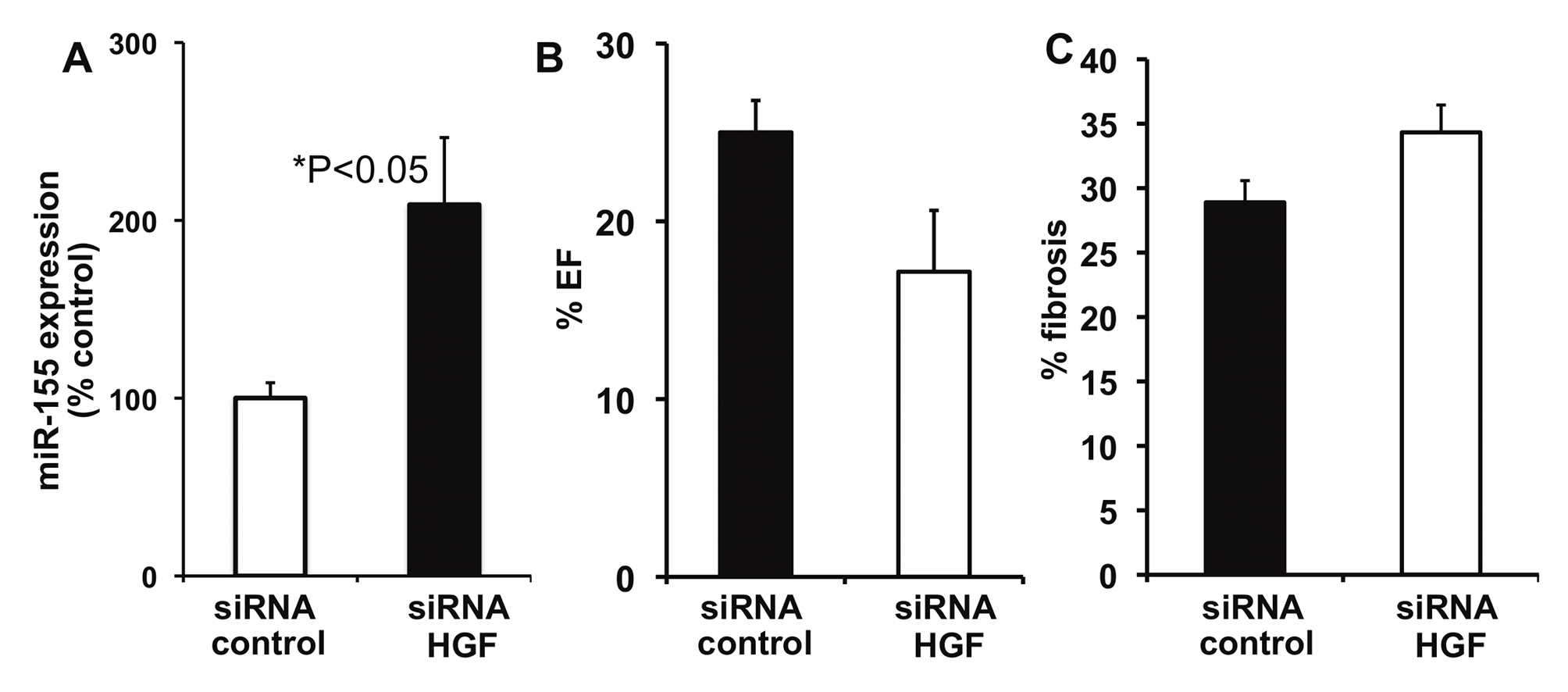

Supplement: Figure S6 — Transplantation of BMPC transfected with siRNA against HGF in mice after MI. (A) miR-155 expression, percent ejection fraction (% EF) (B) and % fibrosis (C) in mice receiving intramyocardial BMPC transfected with siRNA against HGF after MI. *P<0.05 versus control siRNA BMPC-treated MI mice. (TIF) [file pone.0060161.s006.tif]
